# Medical and Philosophical News

**Published:** 1783

**Authors:** 


### SECTION III.

### Medical and Philosophical News.

THE Dutch Society of Sciences at Harlem have propofed the following fubjects of inquiry for a premium of a gold medal. They require require that the author ſhall give, " 1ſt, a ſatiſ-

" factory deſcription of the apparatus that is

" the beſt calculated for making experiments

" on condenſed air in the moſt convenient and

" accurate manner; 2dly, that he ſhall, by ex-

" periments made with ſuch an apparatus, in-

" quire into the action of condenſed air in dif-

" ferent caſes, and, among other things, direct

" his attention to animal life, the growth of

" plants, and the inflammability of different

" ſpecies of air; 3dly, that he ſhall point out

" what conſequences, or what new facts may be

" deduced from his experiments."—The diſſertations on this ſubject are to be written in Dutch,

French, or Latin, and ſent to Mr. C. H. Van der Aa, ſecretary of the Society at Harlem, before the firſt of January 1785.

The Society likewiſe offer a ſimilar premium for the beſt diſſertation on the following ſubject:

" By what rules of conduct, founded on theory,

" and confirmed by experience, will it be poſſi-

" ble to preſerve the health of perſons who, in a

" voyage to the Eaſt Indies, feel the pernicious

" effects of an extreme change of climate and

" manner of living: independent of general

" rules, are there any particular ones to be in-

" dicated, which would vary according to the

" different claſſes of individuals to whom they " might be applicable ?"—Diſſertations on this ſubject will be received till the 31ſt of December 1787.

The Society having received no ſatisfactory reply to their queſtion concerning the different kinds of air, announced in our 1ſt volume, (page 275.) have propoſed it a ſecond time. Candidates for the prize are deſired to ſend their diſſertations to the Secretary before the 1ſt of January 1784.

---

The following Papers (among others, the titles of which we omit, as not falling within the plan of our Journal) have been read at different meetings of the Literary and Philoſophical Society at Mancheſter, ſince the publication of our former account of that inſtitution (vol. 3. p. 91.) —1. An account of a newly-invented machine for impregnating water or other fluids with fixed air; by John Haygarth, M. B. F. R. S.—2. Experiments on the reſpiration of animals, and the changes effected in the air in paſſing through the lungs; by M. Lavoiſier. Tranſlated from the Memoirs of the Academy of Sciences at Paris for 1778, by Mr. Henry, F. R. S.—

3. On

3. On the combuſtion of phoſphorus, and the formation of its acid; by the ſame. Tranſlated by Mr. Henry.—4. Extract of a letter from the Rev. Dr. Griffith of St. Mary Hill, to Dr. Percival, containing an account of the Chineſe, Whang at Tong, lately in London.—5. Obſervations on blindneſs; by Mr. George Bew.—6. A letter to Mr. Maſſey on the formation of ſaltpetre, by Mr. Charles Taylor.—7. On the exiſtence of air in the nitrous acid, and the means of decompoſing and recompoſing that acid; by M. Lavoiſier. Tranſlated by Mr. Henry.—8. Some remarks on fermentation and putrefaction; by Mr. John Wimpey: with ſpeculations by Dr. Percival.—9. An eulogy on the late M. de Haller. Tranſlated from the Memoirs of the Academy of Sciences at Paris, by Mr. Henry.—10. On light and colours, with obſervations on the art of dying; by Mr. John Wilſon.—11. A phyſiological eſſay on melancholy; by James Currie, M. D. of Liverpool.—12. An eſſay to aſcertain the merit of the queſtion reſpecting viſion, formerly diſcuſſed by Mr. Locke and Mr. Molyneux, with ſome remarks on light and colours; by Mr. Wimpey.—13. On the uſe of acids in bleaching, by Dr. Eaſon.—14. Conjectural remarks on the ſymbols or characters employed

 by by the aftronomers to reprefent the feveral planets; and by the chemifts to exprefs the feveral metals. In a letter to Dr. Percival from Martin Wall, M. D. and prælector in chemiftry in the Univerfity of Oxford.—15. Remarks on the knowledge of the ancients; by William Falconer, M. D. F. R. S.—16. On the tea tree; by the Rev. John Foxley, A. M.—17. An effay on the afcent of vapour; by Dr. Eafon.—18. On the natural and chemical hiftory of magnefian earth; by Mr. Henry; and alfo a letter from Mr. de Magellan to Mr. Henry, on the infufibility of that earth.—19. Thoughts on evaporation and electricity; by John Mitchell, M. D.—20. On the regeneration of animal fubftances; by Charles White, Efq. F. R. S.—21. Obfervations on longevity, by Anthony Fothergill, M. D. F. R. S.

---

Dr. Crawford's admirable theory of combuftion and animal heat has lately derived additional weight from the teftimony of M. Lavoifier. This learned French philofopher, who, like our ingenious countryman Mr. Kirwan, fpares no labour or expence in profecuting chemical inquiries, has repeated Dr. Crawford's experiments, and agrees with him perfectly in the refults.

---

The

The *Journal de Medecine* for February 1782 contains two cafes of fchirrous pylorus. The firft is related by M. Amilhon. The patient was a young woman, twenty-two years old. The difeafe began in October 1780 with a pain about the region of the ftomach, which gradually became more violent, and at length the whole inteftinal canal feemed to partake of a painful fenfation. To this fymptom were foon added vomiting and hectic fever. The vomiting at laft became fo conftant as to prevent her taking any nourifhment. She died in June 1781. On diffection the abdominal vifcera were found adhering to each other. The ftomach was leffened and indurated, and a fchirrous tumour of the pylorus, as large as a hen's egg, was feen completely ftopping all communication with the duodenum. The cavity of the thorax contained five or fix pints of water.

The other is the cafe of Count de Lallain, who, at the age of fixty-five, experienced for feveral months lancinating pains and a fenfation of heat at the pit of his ftomach. At firft his appetite was voracious, but it was not long before hectic fever came on, attended with vomiting, and he fometimes brought up food fifteen days after he had taken it. The only thing thing that afforded him any eafe was a folution of gum Arabic in water. He took two or three fpoonfuls of this at a time, and foon afterwards a little broth, which without the previous adminiftration of the gum never failed to excite pain and vomiting. His body was opened after death by Doctors Maloet, Portal, and Bacher, and M. Delafaye.——The omentum was found greatly enlarged and full of hydatids and tubercles. The ftomach was of its ufual fize; but its coats were thickened, and had feveral tubercles in a ftate of fuppuration. The pylorus was confounded in a fchirrous tumour formed of the fubftance of the ftomach. This tumour, which was four inches long and twice as large as an egg, left only a very fmall paffage to the duodenum. Upon cutting into it longitudinally the coats of the ftomach appeared to be more than an inch thick and almoft as hard as cartilage.

---

Extract of a letter from Dr. Lauth, phyfician at Strafburgh, to Dr. Simmons, dated December 16th, 1782.

" Dr. Brand, the city-phyfician-man-midwife " at Leyden, performed the Cæfarian operation " in

" in the courſe of laſt ſummer, with the beſt

" ſucceſs, both the mother and child being alive

" and in perfect health. He made the inciſion,

" not at the *linea alba* as ſome late writers have

" propoſed, but obliquely. He means to pub-

" liſh an account of the caſe. A ſimilar opera-

" tion was performed laſt year at Caſſel, by Dr.

" Stein; but in this caſe the event was leſs for-

" tunate, as the child only was ſaved.——Dr.

" Bonn, profeſſor of anatomy at Amſterdam, is

" preparing a deſcription of the curious collec-

" tion of diſeaſed bones, which Dr. Hovius

" preſented to the college of ſurgeons at that

" place.—Our celebrated profeſſor of chemiſtry,

" Dr. Spielman, has publiſhed within this fort-

" night an excellent *Pharmacopœia Univerſalis*,

" in 4to."

---

Extract of a letter from Dr. Hawes, phyſician to the Surry Diſpenſary, to the editor, dated March 17, 1783.

" In the 3d volume of the London Medical

" Journal the learned and ingenious Dr. Perci-

" val of Mancheſter, recommends to medical

" practitioners the uſe of *Oleum jecoris Aſelli* in

" rheu-

"rheumatic complaints. As every thing that

"comes from the pen of that humane and ex-

"perienced phyſician is deſerving of attention, I

"was deſirous of trying the effects of this oil,

"and accordingly applied to Mr. Jacob of Fiſh-

"ſtreet-hill (druggiſt to the Surry Diſpenſary)

"to procure me a quantity of it from Man-

"cheſter. He very obligingly complied with

"my requeſt, and by means of this new remedy

"I have been enabled to remove ſeveral obſti-

"nate rheumatiſms, which had reſiſted the uſual

"preſcriptions, and where it did not abſolutely

"cure, it afforded greater and more ſpeedy relief

"than guaiacum on any other anti-rheumatic."

---

In the *Journal de Medecine* for February 1783, M. de Vaulevier, phyſician at Fougeres, has publiſhed a very remarkable caſe of a young woman, daughter of a baker of that place, named Briſet. This perſon, who is rather ſhort and fat, with thick, dark hair, and eye-brows, is ſaid to have enjoyed good health till November 1775, when ſhe compleated her 20th year. Since that period her catamenia have ceaſed. For the ſpace of ſix months after this ſuppreſſion of of the menſes ſhe was ſubject to pain in her head, back, and limbs, languor, ſickneſs at ſtomach, and occaſionally to bleeding at the noſe. At the end of that time her health began to mend; and, in proportion as this alteration took place, her chin became covered with a thick, black beard, which obliged her to have recourſe to the raſor. This mark of virility, we are told, is not confined to her face, as the reſt of her body is covered with hair. A mixture of quick lime and orpiment, and various other depilatory applications have been had recourſe to, but hitherto without effect.

---

Dr. Swediar has lately preſented to the Royal Society an intereſting paper on the origin of ambergriſe, a ſubject concerning which various opinions have been entertained. Dr. Swediar, after examining and refuting each of theſe opinions, gives a ſatisfactory account of the origin of this ſubſtance, which appears to be the indurated fæces of the ſpermaceti whale. The black ſpots obſervable in ſpecimens of ambergriſe prove to be the beaks of the *ſepia octopodia* or cuttle-fiſh.

---

We are happy to inform our readers, that the College of Phyſicians have renewed their plan of publiſhing the Medical Tranſactions.

---

The Duc de Chaulnes has lately communicated to the Royal Society a proceſs for obtaining the fuſible ſalt of urine in a ſtate of the greateſt purity and without any loſs.

---

*Works about to be publiſhed.*—1. A new edition of the Edinburgh Diſpenſatory with corrections and additions.—2. A work on the organ of ſmell illuſtrated with very accurate engravings by Dr. Scarpa, profeſſor of anatomy at Modena.—3. A new and improved edition of Dr. Lewis's work on the Materia Medica by Mr. Aikin of Warrington.—4. A new edition of Cronſtedt's Mineralogy, in which all the new diſcoveries will be introduced, by Mr. J. H. de Magellan, F. R. S.——5. M. Bergeret, a French botaniſt, has diſtributed propoſals for printing by ſubſcription a work entitled, " *Phytonomatotechnie* " *univerſelle*, or the art of giving to plants " names derived from their characters; a new

"ſyſtem

" ſyſtem by means of which perſons, without " the aſſiſtance of any book, will be able to " name all the plants that grow on the ſurface " of the globe." The author aſſerts, that by adopting his method, an hundred perſons ſpeaking as many different languages, and reſiding in different parts of the world, will name, and write the names of the ſame plants in the ſame manner as he would write them. He adds, that the principles of his ſyſtem are eaſily learned and retained, and ſo conciſe withal, that thoſe which relate to the different genera may be written on twelve cards. The work is to be printed in numbers, and each number is to contain 12 plates and 24 pages of letter preſs.—6. The Academy of Dijon propoſe to publiſh by ſubſcription a new periodical work in 8vo. entitled, " Nouveaux Memoires ou Cahiers ſe-" meſtres de l'Academie de Dijon, pour la partie " des ſciences & arts." A Number of this work is to appear every ſix months, ſo as to make one volume annually. The terms of the ſubſcription are ſix livres *per annum* for the two numbers delivered at Dijon, or ſeven livres and a half if ſent free of poſtage to any other part of France.

